# Obesity and the risk of catheter-related bloodstream infection: a systematic review and meta-analysis

**DOI:** 10.1186/s13756-022-01166-z

**Published:** 2022-11-12

**Authors:** Yong Wang, Qian Xiang, Jiayu Wu, Na Xiao, Jing Chen

**Affiliations:** 1 Department of Gastrointestinal Surgery, West China Hospital, Sichuan University, Chengdu, China; 2 Department of Healthcare-Associated Infection Control Center, Sichuan Provincial People’s Hospital, University of Electronic Science and Technology of China, Chengdu, China; 3 Chinese Academy of Sciences Sichuan Translational Medicine Research Hospital, Chengdu, 610072 China

**Keywords:** Obesity, Catheter-related bloodstream infection, Meta-analysis

## Abstract

**Background:**

The role of obesity in catheter-related bloodstream infection has been reported in several studies, but it is still controversial. We conducted this meta-analysis to summarize existing evidence to assess the relationship between obesity and the risk of catheter-related bloodstream infection.

**Methods:**

We searched MEDLINE, EMBASE, PubMed and Web of Science for the related studies published before January 2022. Meta-analysis was performed by use of a random-effects model.

**Results:**

A total of 5 articles were included in this meta-analysis. Patients with body mass index  ≥ 25 kg/m^2^ had an increased risk of catheter-related bloodstream infection (OR 1.75, 95% CI 1.38–2.22) in overall analysis. Further analysis indicated that patients with overweight, obesity and severely obesity were all significantly associated with a higher risk of for catheter-related bloodstream infection (OR 1.51 [1.10–2.08], OR 1.43 [1.12–1.82] and OR 2.74 [1.85–4.05], respectively).

**Conclusion:**

This meta-analysis provided evidence that obesity was significantly associated with a higher risk of catheter-related bloodstream infection. Close attention should be paid to the complications and prognosis of obese patients with vascular catheterization in clinical work.

## Introduction

In the past few decades, the prevalence of overweight and obesity has increased rapidly all over the world [1]. This has raised serious public health problems, as overweight and obesity are associated with an increased risk of all-cause mortality [2]. The effects of obesity on the body include a higher risk of cancer, high blood pressure, diabetes, heart disease and death [3]. Available data show that obese people are more likely to develop nosocomial infections than normal weight people [4].

Central catheters are widely used to provide vascular access for injection of drugs, nutrition, blood products and hemodynamic monitoring [5]. Although central catheters provide reliable vascular access, their use have some risks. Of the healthcare-associated infections, catheter-related bloodstream infection (CRBSI) is a worldwide problem and one of the leading causes of death around the world [6]. As an invasive device, the central catheter creates a portal for various microorganisms, and its placement and maintenance are related to excessive infection [7]. Obesity is associated with nosocomial infection, and the association between obesity and intravascular catheter infection is largely considered to exist. Indeed, obesity was found to be an independent risk factor for CRBSI in a large cohort study [8]. A cohort study recently observed a significant correlation between body mass index (BMI) and central-line associated bloodstream infections (CLABSI) [9]. Although the association between obesity and CRBSI is biologically plausible, the results of studies on this relationship are inconsistent.

Studies from different countries have reported the impact of obesity on CLABSI, but there is no systematic analysis of this problem so far. Therefore, we conducted a meta-analysis to summarize the existing evidence on this topic.

## Methods

### Search strategy

We systematically searched MEDLINE, EMBASE, PubMed and Web of Science databases for relevant studies up to January 2022. The following keywords were combined for search: (obesity or overweight or BMI) and (catheter-related bloodstream infections or catheter-related bacteraemia or catheter-related infections or central-line-associated bloodstream infections). We also manually scanned the references lists of each paper to identify additional studies. Two authors reviewed the studies independently, and any disagreement was resolved by discussion and consensus.

### Selection criteria

Studies were included if they met the following criteria: (1) with adults (> 18 years old); (2) investigating the associations between overweight or obesity and catheter-related bloodstream infections; (3) providing hazard ratio (HR) or odds ratio (OR) or relative risk (RR) with 95% confidence interval (95% CI); (4) providing the definition of catheter associated bloodstream infection; (5) with data of relevant results or data required for calculating them. Non-English published studies were excluded. Some studies explored the association between overweight and catheter colonization or catheter-related local infection were also excluded.

### Data extraction

Data were independently extracted and cross-checked by three investigators (JC, YW and QX). The article would be discussed again in case of disagreement. The following data were extracted: the first author, year of publication, the country where the study was conducted, age of individuals, sample size, number of individuals whose weight exceeded the normal range, catheter type, and the definition of outcome. For studies that did not give the mean and standard deviation of any relevant results, we contacted the corresponding authors to provide these values, and included articles that could provide these data.

### Quality assessment

We assessed the methodological quality of all included studies using the Newcastle Ottawa scale (NOS) for the quality assessment of cohort studies and case–control studies [10] and the Cochrane Handbook for randomized controlled trials [11]. The total score of each article was the sum of all items evaluated as positive. For cohort or case‐control studies, 9 points was considered the highest quality.

### Statistical analysis

All statistical analyses were performed with Statistical Software-STATA, version 12.0. The results were summarized as odds ratios (ORs) and 95% confidence intervals (95% CI). The statistical heterogeneity among studies was evaluated by Cochran’s Q test and I^2^ index [12]. P value of less than 0.1 for Q statistic and I^2^ greater than 50% was considered as statistically significant heterogeneity. Due to the heterogeneity found among the included studies, random effect model was applied for all analyses. We performed subgroup analysis according to different BMI. BMI groups were classified as follows: ≤ 18.5 kg/m^2^ (underweight), 18.5–24.9 kg/m^2^ (normal), 25–29.9 kg/m^2^ (overweight), 30.0–39.9 kg/m^2^ (obesity), and ≥ 40.0 kg/m^2^ (severely obesity). The Egger weighted regression method was used to assess publication bias, and P value of less than 0.1 was considered as a statistically significant publication bias.

## Results

### Search results

**Fig. 1:**
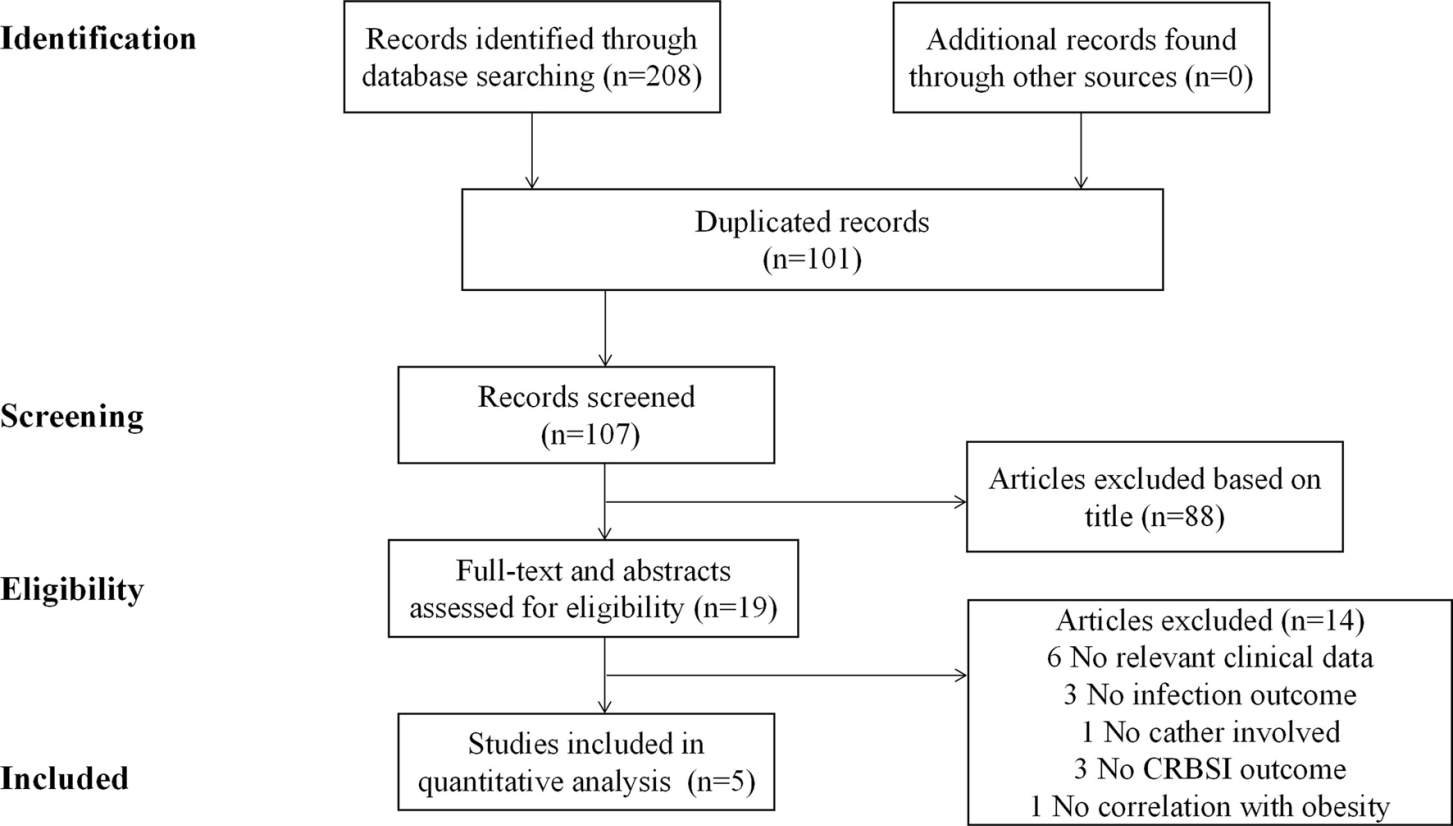
Screening and selection process of studies

We identified 107 non-duplicate articles in the search, and 19 articles were reviewed for full text after screening the titles and abstracts of all articles. Finally, 5 articles were considered eligible and were included for further assessment. Figure 1 showed the literature search process.

### Baseline characteristics

The five included studies were published between 2009 and 2021 [8, 13–16]. The characteristics of the included studies were summarized in Table 1. Of all the studies, three of them were from Europe [13–15] and two were from America [8, 16]. Among the five studies, there were one randomized controlled trial [14], two cohort studies [8, 15], and two case–control studies [13, 16]. The sample sizes were 114–2282 cases. The catheter types included in two articles involved central venous catheter (CVC) [14, 15], two studies involved dialysis catheter [13, 14], and one study involved peripherally inserted central catheter (PICC) [16]. Only one study did not mention the specific type of catheter [8].

**Table 1:** Literature search and study characteristic

Author, year	Country	Study design	Age (years, mean ± SD or medium)	Sample (n)	Patient (n)	Catheter type	Outcome (definition)
Lemaire et al. [13]	France	Case control	62.8 (16–93)	1749	Obese: 143	HD catheter	Bacteremia
Xue et al. [16]	USA	Case control	54 ± 16	114	Overweight or obese: 38	PICC, Tunneled or port	CLABSI
Buetti et al. [14]	France	RCT	65.2 (55.7–74.4)	2282	Obese: 1880 Morbidly obese: 402	CVC, AC and DC	CRBSI
Schalk et al. [15]	Germany	Cohort	NA	1046	Overweight: 405 Obese: 294	CVC	CRBSI
Dossett et al. [8]	USA	Cohort	NA	2037	Overweight: 615 Obese: 494 Severely obese: 192	Not mentioned	CRBSI

*HD* hemodialysis; *CVC* central venous catheter; *PICC* peripherally inserted central catheter; *AC* arterial catheter; *DC* dialysis catheter; *CLABSI* central-line-associated bloodstream infections; *CRBSI* catheter-related bloodstream infections

### Study quality

The randomized controlled trial [14] had a low risk of bias for all items (Table 2). Among the cohort studies [8, 15], one study scored 6 stars [15], and another scored 7 stars [8] (Table 3). The two case–control studies [13, 16] scored 5 stars (Table 4).

**Table 2:** Cochrane review for risk of bias

Study	Random sequence generation (selection bias)	Allocation concealment (selection bias)	Blinding of participants and personnel (performance bias)	Blinding of outcome assessment (detection bias)	Incomplete outcome data (attrition bias)	Selective reporting (reporting bias)	Other bias
Buetti et al. [14]	Low	Low	Low	Low	Low	Low	Low

**Table 3:** NOS criteria for cohort study

Study	Representativeness of the exposed cohort	Selection of the non-exposed cohort	Ascertainment of exposure	Demonstration that the outcome of interest was not present at the start of the study	Comparability of cohorts on the basis of the design or analysis	Assessment of outcome	Was follow-up long enough for outcomes to occur	Adequacy of follow-up of cohorts	Total quality scores
Schalk [15]	☆	☆	\	☆	☆	☆	☆	\	6
Dosset al. [8]	☆	☆	☆	☆	☆	☆	☆	\	7

**Table 4:** NOS criteria for a case–control study

Study	Is the case definition adequate?	Representativeness of the cases	Selection of controls	Definition of controls	Comparability of cases and controls on the basis of the design or analysis	Ascertainment of intervention	Same method of ascertainment for cases and controls	Non-response rate	Total quality scores
Lemaire et al. [13]	☆	☆	\	☆	\	☆	☆	\	5
Xue et al. [16]	☆	☆	\	☆	\	☆	☆	\	5

### Catheter-related bloodstream infection

One study reported a positive association between obesity or severe obesity and catheter-related bloodstream infection [8]. In two studies, BMI > 25 kg/m^2^ was not related [13, 16]. Compared to normal weight, overweight patients had higher CRBSI rate but obese patients did not in Schalk’s study [15]. Buetti’s study only included obese patients, and the study reported that BMI ≥ 40 kg/m^2^ had higher risk for CRBSI [14].

**Fig. 2:**
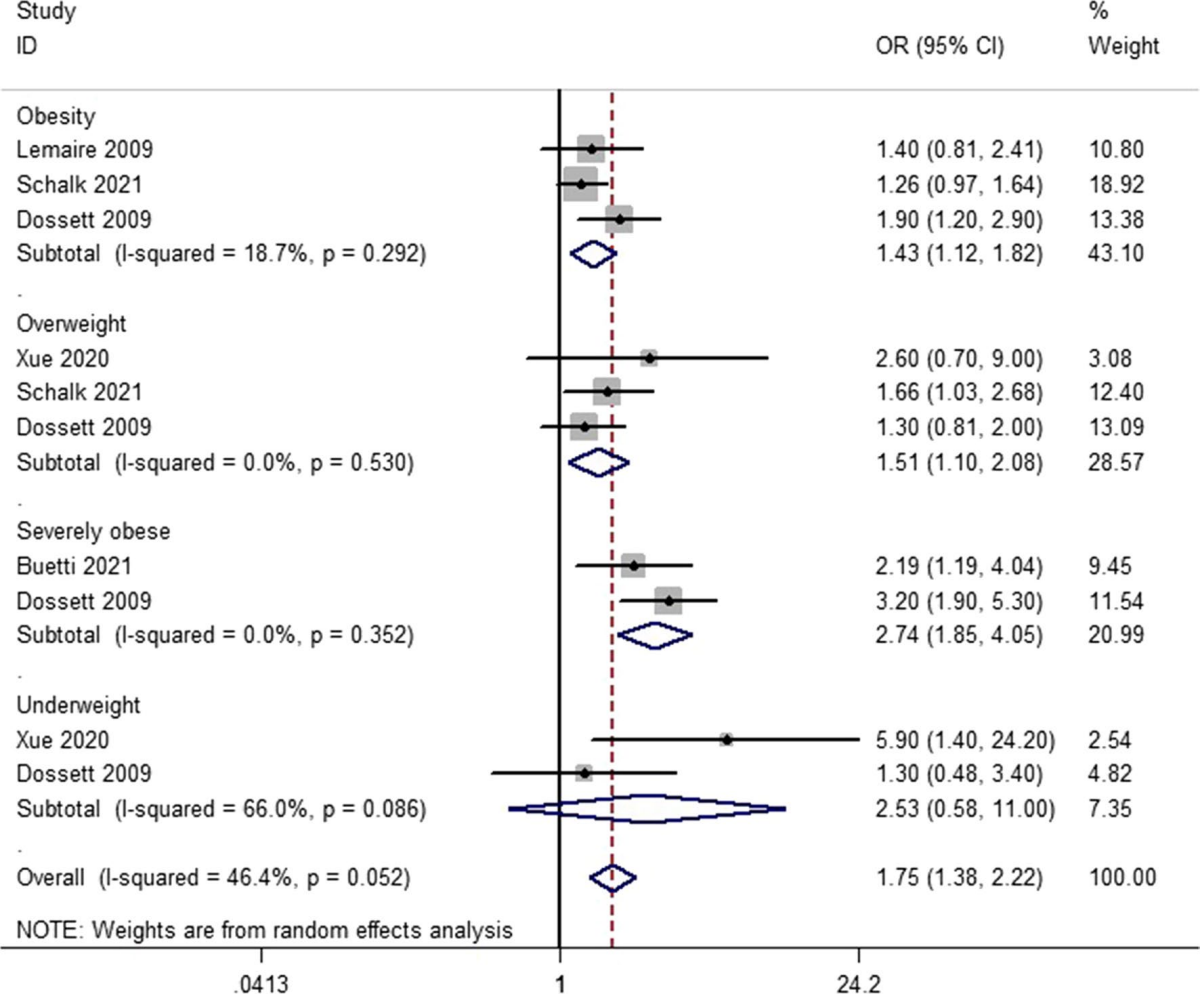
Forest plot for catheter-related bloodstream infection in different body mass index (BMI)

The overall meta-analysis showed that patients with BMI higher than normal had an increased risk of CRBSI (OR = 1.75; 95% CI 1.38, 2.22) (Fig. 2). Further analysis was conducted by BMI categories (Fig. 2). In the three studies [8, 13, 15] using the obesity data could be pooled and the OR for CRBSI was 1.43; 95% CI 1.12, 1.82; I^2^ = 18.7%. For the three studies [8, 15, 16] pooled on the basis of overweight data, the OR for CLABSI was 1.51; 95% CI 1.10, 2.08; I^2^ = 0% and for two studies [8, 14] using the severely obesity data, the OR was 2.74; 95% CI 1.85, 4.05; I^2^ = 0%. In the underweight subgroup, it did not demonstrate a significant association. However, the heterogeneity of this group was high due to the few data.

### Publication bias

Publication bias was assessed using the Egger weighted regression method, and no publication bias was found in the analysis (*P* = 0.455).

## Discussion

This was the first meta-analysis to evaluate the association between obesity and the risk of catheter-related bloodstream infection. Previous data indicate an association between obesity and infection [17]. Persons with obesity are more prone to develop nosocomial infection, surgical site, skin and soft tissue infection, bloodstream infection, and urinary tract infection [18]. Nosocomial bloodstream infections are usually associated with the use of intravascular device, such as CVCs [19]. Indeed, we found the association between obesity and intravascular catheter infection. In our meta-analysis, we found that patients with overweight, obesity or severely obesity had an increased risk for CRBSI. We also found that there was no significant association between underweight and CRBSI.

Obesity related immune system disorders, decreased cell-mediated immune response, obesity related comorbidity and respiratory dysfunction have been considered as possible mechanisms of infection [17, 20]. There are several explanations for the potential association between obesity and higher risk of CRBSI. One of them is that due to the loss of physical markers and the long distance from the superficial structure to central vessel in obese patients, the placement of the central catheter is more difficult [21, 22]. In view of this, clinicians may hesitate to replace catheters in case of suspicion of infection in obese patients, thereby increasing the risk of CRBSI. Another possible explanation is that the increase of sweating tendency in obese patients may cause the dressing of the catheter disruption. Previous data showed that dressing interruption was an important risk factor for intravascular catheter-related infection [23].

Furthermore, obesity has a significant impact on immune surveillance [24]. Adipose tissue regulates the interaction between immune system and adipose tissue by secreting adipokines [25]. Obesity violates the balance system of adipocytes and immune cells, and then interfere with the immune surveillance system, leading to immune response disorders [24, 25]. At the same time of immune response disorder, puncture can destroy the vascular barrier and increase the chance of pathogen invasion, thus increasing the risk of infection. In addition, the blood vessels of obese patients are not easy to puncture, so femoral vein may be selected during catheterization. Femoral vein may be a risk factor for infectious complications [26].

In our study, heterogeneity was observed through the result of inconsistency test (I^2^). The main source of heterogeneity was that there were few studies included. There are differences in obese patients included in different studies. There were differences in the definition of obesity in these studies, but our final analysis was based on BMI provided from each study.

A few limitations of our study should be taken into consideration. Firstly, there were differences in the definitions of intravascular catheter-related bloodstream infection in these studies. The definition of CRBSI in few studies required not only positive blood culture, but also the cultivation of the same pathogen at the tip of the catheter. This definition was stricter, so it was possible to miss some cases of infection, which may influence the accuracy of the overall results. Secondly, weight and height measurements were not standardized in different studies. Thirdly, although the original studies included attempted to control other risk factors, residual unknown confounding factors might also affect the results. Finally, the number of studies included in our analysis was small. Further large sample and multicenter studies are needed to clarify the relationship between obesity and catheter-related bloodstream infection.

In conclusion, this meta-analysis suggested that overweight, obesity and severely obesity were significantly associated with a higher risk of catheter-related bloodstream infection. Given that obesity is common, and associated with infection and other complications in the population, efforts to understand the impact of obesity on patients’ infection and long-term outcomes after infection should be a research focus.

## Data Availability

The data sets used and/or analyzed in this study can be obtained from the corresponding authors upon reasonable request.
